# Le HELLP syndrome: à propos de 61 cas et revue de la littérature

**Published:** 2012-02-20

**Authors:** Nisrine Mamouni, Hakima Bougern, Ali Derkaoui, Karima Bendahou, Samira Fakir, Chehrazad Bouchikhi, Hikmat Chaara, Abdelaziz Banani, Moulay Melhouf Abdelilah

**Affiliations:** 1 Service de gynécologie–obstétrique, CHU Hassan II, Fès, Maroc; 2 Service de réanimation, CHU Hassan II, Fès, Maroc; 3 Laboratoire d’épidémiologie clinique et d’analyse statistique, Faculté de médecine et de pharmacie de Fès, Maroc

**Keywords:** HELLP syndrome, Prééclampsie, Grossesse, Maroc

## Abstract

**Introduction:**

L’objectif de ce travail etait d’étudier et comparer les paramètres épidémiologiques, cliniques, paracliniques et évolutifs des patientes présentant un HELLP syndrome complet et incomplet. (Hemolysis, Elevated Liver enzymes, Low Platelets count).

**Méthodes:**

Une enquête rétrospective sur la période du 1er janvier 2005 au 31octobre 2008 et incluant tous les cas de HELLP syndrome colligés au service de Gynécologie obstétrique du CHU HASSAN II de Fès, incluant toutes les patientes ayant présenté un HELLP syndrome. Les patientes ont été classées en deux groupes en fonction de la forme du HELLP syndrome (complète ou incomplète).

**Résultats:**

61 patientes ont été incluses, dont 29 ont présenté un HELLP syndrome complet (groupe 1) et 32 ont présenté une forme incomplète de ce syndrome (groupe 2). La moyenne d’âge était de 29,7 ± 7 ans pour le groupe 1 et de 28,5 ± 7 ans pour le groupe 2.La gestité moyenne était de 2,8, Pour le groupe 1et de 2,5 pour le groupe 2.Les complications maternelles ont été notées chez 68,9% des patientes avec HELLP complet et 53% avec HELLP incomplet. On a recensés trois cas de décès maternel dans le groupe HELLP complet contre aucun cas dans la forme incomplète.

**Conclusion:**

D’après les résultats de notre étude, le HELLP complet n’expose pas les patientes à un risque plus élevé de complications maternelles que dans la forme incomplète.

## Introduction

L’association d’une hémolyse, d’une cytolyse hépatique, et d’une, thrombopénie au cours de la prééclampsie sévère est connue depuis de nombreuses années [1]. C’est à Weinsteïn [2] en 1982 que revient le mérite d’avoir individualisé cette pathologie en créant l’acronyme HELLP syndrome (Hemolysis, Elevated Liver enzymes, Low Platelet count).

Le but de notre étude est de comparer les paramètres épidémiologiques, cliniques, paracliniques et évolutifs des patientes présentant un HELLP syndrome complet et incomplet.

## Méthodes

Nous avons réalisé une enquête rétrospective sur la période du 1er janvier 2005 au 31 octobre 2008 et incluant tous les cas de HELLP syndrome colligés au service de Gynécologie obstétrique du CHU HASSAN II de Fès. Nous avons réparti les patientes en deux groupes. Le groupe 1 (les patientes présentant un HELLP complet) et le groupe 2 (les patientes avec HELLP incomplet). Les critères diagnostiques du HELLP syndrome complet retenus pour notre étude sont ceux qui ont été énoncés par Sibaï [3] , associant une thrombopénie de moins de 100000 plaquettes par mm^3^, une cytolyse hépatique marquée par une élévation des enzymes hépatiques (TGO et TGP), ainsi qu’une hémolyse intravasculaire mise en évidence par une baisse du taux d’hémoglobine (3], décrit par l’existence de : EL (cytolyse isolée), HEL (hémolyse avec cytolyse), HLP (hémolyse et thrombopénie), LP (thrombopénie isolée).

Nous avons relevé l’âge des patientes de chaque groupe, les conditions du diagnostic du HELLP syndrome (pré ou post-partum), leur provenance d’une autre maternité, l’âge gestationnel, la cinétique des anomalies biologiques, les signes cliniques à l’entrée dans le service, l’indication et le mode d’accouchement, le délai entre le moment de la prise en charge et celui de l’accouchement et l’existence de complications.

Le bilan biologique à l’admission comprenait: la protéinurie des 24 heures, l’ionogramme sanguin, la fonction rénale (créatinine et urée sanguine), le bilan de coagulation (TP, TCA), le dosage de l’acide urique et si le taux de plaquettes était très bas, nous avions procédé à la recherche de signes de coagulation intra vasculaire disséminée (CIVD). Une corticothérapie était débutée si le terme de la grossesse était inférieur à 34 semaines d’aménorrhée (SA) et si un traitement de type conservateur au niveau obstétrical était décidé. Lorsqu’une attitude conservatrice a été retenue, la surveillance maternelle a été effectuée par le contrôle quotidien du bilan biologique. Le contrôle du bien être fœtal a été basé sur la réalisation d’une échographie Doppler.

Les dossiers des nouveau-nés ont été analysés en retenant leur poids de naissance et l’existence d’un retard de croissance intra-utérin.

Le but de l’étude est de comparer les deux formes de HELLP syndrome (complète et incomplète) afin de dégager les facteurs pronostic dont dépend l’évolution dans les deux groupes.

Les résultats ont été exprimés en nombre, en pourcentage ou en moyenne ± écart-type. Pour l’analyse univariée, les variables quantitatives ont été comparées par le test t de Student et les variables qualitatives par le Chi2 ou le test exact de Fisher. Les statistiques ont été effectuées à l’aide du logiciel épi info.

## Résultats

Durant la période de l’étude, 22450 patientes ont accouché dans le service de la maternité de l’hôpital universitaire de Fès. Un HELLP syndrome a été retrouvé chez 61 patientes. L’incidence estimée de ce syndrome était donc de 0,27 %, soit de 1 cas pour 361 grossesses. L’incidence du HELLP chez les patientes préeclamptiques admises durant la même période est de 15,21%. Aucune des patientes n’a bénéficié d’un suivi prénatal au sein de notre formation et 67% de nos patientes ont été référées des maternités périphériques (Taounate, Tissa, Sefrou, Boulmane...).

**Figure 1:**
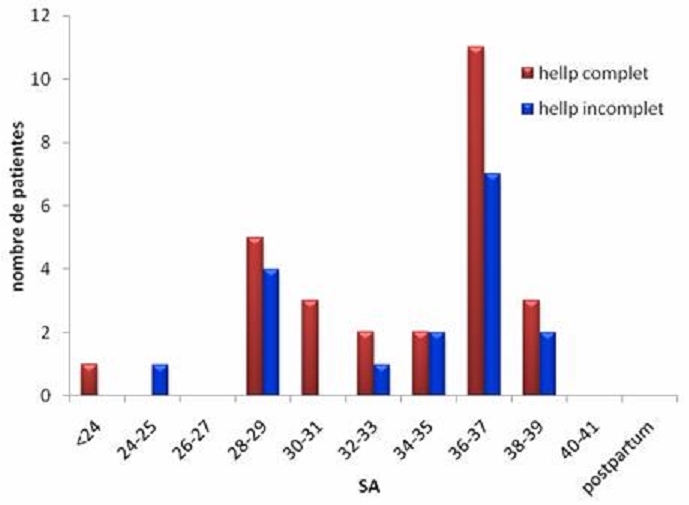
Répartition des patientes en fonction de l’âge gestationnel

Parmi les 61 cas de HELLP syndrome, 29 ont présenté une forme complète (47,54 %), et 32 avaient un HELLP incomplet (52 ,45 %). La moyenne d’age était de 29,7 ± 7 ans pour le groupe 1 (HELLP complet) et de 28,5 ± 7 ans pour le groupe 2(HELLP incomplet). Il n’y avait pas de différence significative entre les deux groupes. Pour les vingt-neuf patientes du groupe 1, la gestité moyenne était de 2,8 (se répartissant de 1 à 10) et la parité moyenne de 2. Pour les 32 patientes du groupe 2, la gestité moyenne était de 2,5 (de 1 à 5) et la parité moyenne de 1,7. L’âge gestationnel moyen au moment de l’hospitalisation dans notre maternité était de 33,6 ± 4,8 SA pour le groupe 1 et de 33 ,3 ± 5 pour le groupe 2. La répartition des patientes en fonction de l’âge gestationnel est représentée sur la Figure 1.

Le taux d’incidence du HELLP syndrome du post partum de notre série était de 14,7 % (9 patientes sur 61). 4 patientes du (groupe 1) et 5 du (groupe 2). Les différents signes cliniques observés à l’entrée sont récapitulés dans le Tableau 1.

**Tableau 1:** Signes cliniques à l’admission. Les variables qualitatives sont présentées en effectif (pourcentage)

Signes cliniques	HELLP complet	HELLP incomplet	p
HTA	85,2%	100,0%	0,16
PROTEINURIE positive	91,7%	94,1%	1,00
Céphalées (présence)	100 %	80%	1,00
Barres épigastriques (présence)	25%	20%	1,00
Nausées vomissement (présence)	50 %	50 %	0,4
ictère	3,8	13,3	0,3

HTA : Hypertension artérielle.

Les éléments biologiques constitutifs du HELLP syndrome sont résumés dans le Tableau 2. Pour les deux groupes, le délai moyen entre le diagnostic et l’accouchement était de 12 heures.

**Tableau 2:** Paramètres biologiques à l’admission. Les variables quantitatives sont présentées sous forme de moyenne ± écart-type, les variables qualitatives en effectif (pourcentage)

Paramètres biologiques	HELLP complet (1)	HELLP incomplet (0)	p
Taux d’hémoglobine moyen	8,764	8,944	0,74
Taux de bilirubine totale	19,95 (3,64)	9,54 (5,62)	< 10-4
LDH moyen	665,14	238,20	<10-3
Taux de plaquettes moyen (diagnostic)	80827,59	131235,29	0,02
Taux de GOT moyen (diagnostic)	214,48	151,76	0,15

Les complications maternelles sont résumées dans le Tableau 3. Les complications maternelles ont été notées chez 74 % de nos patientes, Ces complications ont été notées chez 71,4 % des patientes du (groupe 1) et 77,3 % des patientes du (groupe 2).la différence entre le pourcentage des femmes qui ont fait des complications ne diffère pas entre le groupe qui avait un Hellp complet ou incomplet (p =0,751). 46,6 % des complications maternelles ont été notées chez les patientes ayant présenté le HELLP syndrome en post-partum. Vingt-sept (44%) patientes ont présenté des crises d’éclampsie, ceci chez 59% des patientes du groupe 1 et 50% des patientes du groupe 2. Sept de ces crises se sont produites lors de HELLP syndrome du post-partum.

**Tableau 3:** Complications maternelles. Les variables qualitatives sont exprimées en pourcentage

Complications maternelles	HELLP complet	HELLP incomplet	p
Eclampsie (présence)	59,1%	50,0%	0,58
Hématome sous capsulaire foie (1)	21,4%	0,0%	0,06
IRA	38,5%	45,5%	0,7
PRES	11,1 %	0%	0,3
OAP (1)	0%	55,6%	0,004
HRP	3,6%	13,3%	0,27
CIVD (1)	40,9%	0,0%	0,06

IRA: Insuffisance rénale aigue ; OAP: œdème aigue du poumon, CIVD: coagulation intravasculaire disséminée ; HRP: Hématome retro-placentaire ; PRES: leucoencéphalopathie postérieure réversible

Dix (16 %) patientes ont présenté des troubles de la coagulation à type de CIVD. Toutes ces patientes avaient un HELLP complet (Groupe 1) et ayant présenté d’autres complications que la CIVD: Hématome Rétro Placentaire (HRP), et/ou éclampsie, et/ou insuffisance rénale, et/ou hématome sous capsulaire du foie.

21% des patientes du Groupe 1 ont présenté un hématome sous capsulaire du foie, alors qu’aucune patiente du groupe 2 n’a présenté cette complication (P=0,06). Le diagnostic a été fait à chaque fois par échographie abdominale. Cet examen a été réalisé chez 32 patientes en raison de douleurs épigastriques très importantes. Une patiente a bénéficié en plus de l’échographie d’un scanner abdominal (post-partum). Il n’y a eu aucun cas de rupture hépatique.

Trois patientes ont présenté une leucoencéphalopathie postérieure réversible (PRES) (0,04%).ces patientes avaient toutes un HELLP complet (groupe 1). 68 % des patientes du groupe 1 et 53 % des patientes Du groupe 2 ont accouché par césarienne (par incision de Pfannenstiel). Les caractéristiques des enfants à la naissance sont récapitulées dans le Tableau 4. Nous avons déploré trois cas de mortalité maternelle appartenant tous au groupe1.

**Tableau 4:** Caractéristiques des enfants à la naissance. Les variables quantitatives sont présentées sous forme de moyenne ± écart-type, les variables qualitatives en effectif (pourcentage)

	HELLP complet	HELLP incomplet	p
Prématurité (1)	39,1 %	50,0%	0,72
Age gestationnel moyen	33,18 semaines A	33,76 semaines A	0,69
poids	1987,5	1815,0	0,7
prématurité	39,1	50,0	0,4
RCIU	19,0	25,0	0,5

RCIU : retard de croissance intra-utérine

La première patiente âgée de 23 ans, primigeste, admise dans un état d’éclampsie sur une grossesse à terme. Son bilan biologique a objectivé un HELLP complet (le taux de plaquettes à 55000/mm^3^). L’extraction fœtale urgente par voie haute. La patiente a été intubée pendant 24 H ayant présenté des troubles neurologiques après son extubation. Un scanner cérébral réalisé objectivant une leucoencéphalopathie réversible. La patiente est décédée après 13 jours d’hospitalisation dans le cadre d’une encéphalopathie post anoxique.

La deuxième patiente âgée de 33 ans, admise pour prise en charge d’une hypertension artérielle sévère sur une grossesse estimée à 35 SA .le bilan biologique initial a été normal. L’extraction fœtale réalisée par voie haute En post opératoire immédiat la patiente a présenté une instabilité hémodynamique associée à un ballonnement abdominal. Un contrôle des plaquettes est revenu à 25000/ mm^3^ avec un TP=10 %. Une échographie abdominale réalisée a objectivé un épanchement intrapéritonéal de grande abondance. Une laparotomie a été réalisée mettant en évidence un hémopéritoine de grande abondance suite à la rupture d’un hématome sous capsulaire du foie. La patiente a bénéficié d’un lavage péritonéal avec mise en place d’un packing. Elle est décédée après une heure en post opératoire dans un tableau d’état de choc hémorragique réfractaire. La troisième patiente est âgée de 34 ans, admise pour crise d’éclampsie sur grossesse estimée à 26 SA. Le bilan a été normal .un traitement conservateur a été de mise. Vu la survenue d’une récidive de la crise d’éclampsie après vingt-quatre heures avec l’installation d’un HELLP complet et de trouble de la coagulation (CIVD), une césarienne a été décidée pour sauvetage maternel donnant naissance à un mort-né. Après vingt heures en post opératoire, la patiente a présenté un ballonnement abdominal et une instabilité hémodynamique avec à l’échographie la présence d’un épanchement intraperitoneal de grande abondance. Une laparotomie fut réalisée objectivant un hématome sous capsulaire hépatique fissuré d’où la mise en place d’un packing en inter hépato-diaphragmatique. La patiente est décédée vingt-quatre heures plus tard dans un tableau d’état de choc hémorragique avec défaillance multi-viscérale.

## Discussion

Selon Sibaï et al. [3], la patiente typique présentant Un HELLP syndrome serait de race blanche, multipare, âgée de plus de 25 ans. Cette pathologie se déclarerait avant la fin de la grossesse, à un terme généralement inférieur à 36 SA. Dans la plupart des grandes séries de la littérature [4,5], les primigestes concernées par cette pathologie seraient 1,5 à 2 fois plus nombreuses que les multigestes. Cette tendance n’est pas retrouvée dans notre série où 41 ,3 % (HELLP complet) et 37,5 % (HELLP incomplet) des patientes sont primigestes. Pour Sibaï et al. [6], le terme moyen d’apparition du HELLP syndrome se situait entre 27 et 36 SA dans 71 % des cas, était inférieur à 27 SA dans 11 % des cas, et était supérieur à 37 SA dans 18 % des cas. Ces chiffres sont tout à fait semblables à ceux retrouvés dans notre étude où l’âge gestationnel moyen était de 33,18 SA pour Le groupe du HELLP complet et de 33,76 SA pour le groupe du HELLP incomplet (Tableau 4). Le HELLP syndrome du post-partum concerne les patientes qui ont présenté pour la première fois en postpartum les manifestations cliniques et biologiques de cette pathologie. Elles représentaient 30 % des cas de la série de Sibaï et al. [6]. Ces patientes présenteraient un risque plus élevé d’insuffisance rénale et d’œdème aigu pulmonaire. La difficulté est de savoir si cette pathologie diagnostiquée après l’accouchement ne débute pas en fait dans le pré-partum. On peut aussi se demander si le HELLP syndrome du post-partum n’a pas une physiopathologie différente de celle du HELLP syndrome classique, ce qui pourrait expliquer son développement accentué malgré la délivrance réalisée. Neuf patientes (14,7%) de notre série ont présenté un HELLP syndrome du post-partum (4 patientes du groupe HELLP complet et 5 du groupe HELLP incomplet). Comme pour Sibaï et al.[6], ces patientes ont accouché près du terme. Toutes ces patientes, ont présenté une ou plusieurs complications graves (CIVD, éclampsie, insuffisance rénale, œdème aigu du poumon, leucoencéphalopathie postérieure réversible). En analyse univariée, la survenue du HELLP au cours du post-partum est un facteur de risque de survenue de complication maternelle dans notre étude (p=0 ,04).La sévérité du HELLP syndrome dans le post-partum est peut-être due à sa découverte qui ne se fait qu’au stade de la complication.

Aucun signe clinique n’est pathognomonique du HELLP syndrome. Trois signes cliniques doivent cependant attirer l’attention : l’HTA, les céphalées et la barre épigastrique. Martin et al. [7] considère que la thrombopénie est le critère pronostique le plus important du HELLP Syndrome et serait pour lui prédictive du risque de récidive ultérieure de cette pathologie. Par ailleurs, plus la thrombopénie est importante, plus le délai d’accouchement sera bref, plus les complications seront nombreuses et plus le taux de transfusion sera élevé. Pour Audibert et al. [9], il est important de corréler la thrombopénie avec les autres paramètres biologiques du HELLP syndrome. Les patientes présentant un HELLP incomplet présentent moins de complications dans sa série, comme c’est le cas dans notre série.

La morbidité maternelle du HELLP syndrome est plus importante lorsqu’on la compare à celle de la pré-éclampsie simple Martin et al. [11] notent que le risque de complications sévères n’est pas annulé immédiatement après l’évacuation du contenu utérin (la preuve en est dans les HELLP syndromes du post-partum). Le taux de mortalité et la fréquence des complications maternelles de notre série ne diffère pas de ceux des grandes séries de la littérature [6,11,12].Les complications maternelles ont été notées chez 74% de nos patientes, 68,9% des patientes avec HELLP complet ont eu une ou plusieurs complications contre 53% des patientes avec HELLP incomplet, la différence n’est pas statistiquement significative (p=0,75) .

Weinsteïn [13] rapporte deux décès sur 57 cas (3,5 %) : un décès par anémie sévère avec crise d’éclampsie et un autre par lésions hémorragiques péritonéales. Dans notre série, le taux de mortalité était de 0,04 % (3 patientes sur 61).

La Coagulation Intra Vasculaire Disséminée (CIVD) est la complication la plus fréquemment retrouvée dans les différentes séries de la littérature [3,6,13,14]. Dans notre série, cette complication est survenue principalement chez les patientes ayant un HELLP complet et ceci chez 40,9% d’entre elles (p = 0,06).

Martin et al. [17] ne retrouvaient pas d’augmentation des crises d’éclampsies quand les troubles biologiques sont les plus sévères. Vingt-sept patientes de notre série ont présenté une crise d’éclampsie sans différence statistique entre les deux groupes. A noter par contre, une fréquence importante de cette complication dans le post-partum (7/9), ce qui est retrouvé dans la série de Roussillon [18] et pas dans les autres séries de la littérature.

L’hématome sous capsulaire du foie est une complication spécifique du HELLP syndrome. Son incidence est de 0,9 % dans la série de Sibaï et al. [6]. Sibaï et al. [6] rapportent un décès sur quatre cas d’hématomes rompus. Six patientes de notre série ont présenté un hématome sous capsulaire du foie sans rupture hépatique .Toutes ces patientes appartenaient au groupe du HELLP complet.

Un hématome rétro-placentaire survenait dans 20 % des cas de la première série de Sibaï et al. [15] et dans 16% des cas de sa série de 1993 [6].Dans notre série, cette complication est survenue dans 3,6 % des cas de HELLP complet et 13,3% des cas de HELLP incomplet.

Le traitement et la prise en charge du HELLP syndrome reste un sujet très controversé [14,18–22]. La plupart des auteurs [2,3,6,15,23] recommandent un traitement non conservateur par interruption immédiate de la grossesse et ceci quel que soit l’âge gestationnel. L’autre alternative est un traitement conservateur : un tel traitement n’a pas lieu d’être mis en place après 32 SA. Cependant, lorsque le terme est inférieur à 32 SA et lorsque le taux de plaquettes est > 50 000/mm^3^, certains auteurs proposent, en l’absence de souffrance fœtale aiguë et de complications maternelles, de différer le moment de l’extraction fœtale. Ceci améliorerait le pronostic fœtal [24,25], le pronostic maternel et permettrait un transfert in utero vers un centre de niveau 3.

## Conclusion

Le HELLP syndrome est une forme particulière de pré-éclampsie sévère. C’est un signe de gravité indiscutable de la maladie en termes de mortalité et de morbidité maternelle. Notre étude rétrospective comparant deux groupes de patientes ayant un HELLP complet ou incomplet démontre que la forme complète de ce syndrome n’expose pas les patientes à un risque plus élevé de complications maternelles (hémorragiques, neurologiques, pulmonaires, rénales).
